# The endoplasmic reticulum unfolded protein response – homeostasis, cell death and evolution in virus infections

**DOI:** 10.1093/femsre/fuab016

**Published:** 2021-03-25

**Authors:** Vibhu Prasad, Urs F Greber

**Affiliations:** Department of Molecular Life Sciences, University of Zurich, Winterthurerstrasse 190, 8057 Zurich, Switzerland; Department of Molecular Life Sciences, University of Zurich, Winterthurerstrasse 190, 8057 Zurich, Switzerland

**Keywords:** endoplasmic reticulum unfolded protein response, virus-induced cell stress, cell death, homeostasis, evolution, stress response

## Abstract

Viruses elicit cell and organismic stress, and offset homeostasis. They trigger intrinsic, innate and adaptive immune responses, which limit infection. Viruses restore homeostasis by harnessing evolutionary conserved stress responses, such as the endoplasmic reticulum (ER) unfolded protein response (UPR^ER^). The canonical UPR^ER^ restores homeostasis based on a cell-autonomous signalling network modulating transcriptional and translational output. The UPR^ER^ remedies cell damage, but upon severe and chronic stress leads to cell death. Signals from the UPR^ER^ flow along three branches with distinct stress sensors, the inositol requiring enzyme (Ire) 1, protein kinase R (PKR)-like ER kinase (PERK), and the activating transcription factor 6 (ATF6). This review shows how both enveloped and non-enveloped viruses use the UPR^ER^ to control cell stress and metabolic pathways, and thereby enhance infection and progeny formation, or undergo cell death. We highlight how the Ire1 axis bypasses apoptosis, boosts viral transcription and maintains dormant viral genomes during latency and persistence periods concurrent with long term survival of infected cells. These considerations open new options for oncolytic virus therapies against cancer cells where the UPR^ER^ is frequently upregulated. We conclude with a discussion of the evolutionary impact that viruses, in particular retroviruses, and anti-viral defense has on the UPR^ER^.

## Teaser

Although viruses trigger robust stress responses and innate and adaptive host defense, we reveal that a large spectrum of enveloped and non-enveloped viruses activate homeostasis restoring processes, especially the endoplasmic reticulum unfolded protein response, which promotes virus propagation and survival.

## ABBREVIATIONS

AdV: Adenovirus
AARE: Amino acid response element (biding sites for ATF4 transcription factor)
AAV: Adeno-associated virus
AdV: Adenovirus
ASFV: African swine fever virus
ATF4: Activating transcription factor 4
ATF6: Activating transcription factor 6
Bak: Bcl-2 homologous antagonist or killer
Bax: Bcl-2 associated X-protein
Bcl2: B-cell lymphoma 2
Bim: Proapoptotic protein Bcl-2 like protein 11 (Bcl2L11)
BiP/Grp78: Binding immunoglobulin protein/Glucose regulated protein 78
BVDV: Bovine viral diarrhea virus
CVA16: Coxsackievirus A16
CVB3: Coxsackievirus B3
Cnx: Calnexin
Crt: Calreticulin
C/EBP: CCAAT/-enhancer-binding protein
CD4: Cluster of differentiation 4 glycoprotein
CHOP: CCAAT/-enhancer-binding protein homologous protein
CMV: Cytomegalovirus
COPII: Coatomer protein 2
CREBH: Cyclic adenosine monophosphate (cAMP)-responsive element-binding protein H
DENV: Dengue virus
DDR: DNA damage response
DnaK: Bacterial chaperone Hsp70
EDEM: ERAD enhancing α-mannosidase-like proteins
eIF2α: Eukaryotic translation initiation factor 2 subunit1
ERAD: ER-associated degradation
ERdj4: ER-localised J-protein 4
ERSE: ER-stress response elements
GADD34: Growth arrest and DNA damage-inducible protein 34
HCMV: Human cytomegalovirus
HCV: Hepatitis C virus
HIV: Human immunodeficiency virus
Hsp: Heat shock protein
HSV: Herpes simplex virus
IAV: Influenza A virus
IFNAR1: Interferon alpha or beta receptor subunit 1
IFNß: Interferon-ß
IκB: Inhibitor of κB
IKK: IκB kinase
IL-6: Interleukin-6
Ire1α: Inositol-requiring enzyme 1 alpha
ISR: integrated stress response
ISRIB: ISR inhibitor
JeV: Japanese encephalitis virus
JNK: c-Jun N-terminal kinases
MHC-I: Major histocombatibility factor I
MHV: Mouse gammaherpes virus
NADPH: Nicotinamide adenine dinucleotide phosphate (reduced form)
NF-κB: Nuclear factor kappa light-chain-enhancer of activated B cells
NOX2: NADPH oxidase 2
ORF: open-reading frame
PERK: Protein kinase activated by double stranded RNA (PKR)-like ER kinase
PMV: Paramyxo simian virus
PP1: Protein phosphatase 1
RSV: Respiratory syncytial virus
RIDD: Regulated Ire1-dependent decay of mRNA
ROS: Reactive oxygen species
RPS: Ribosomal protein subunit
SARS-CoV: Severe acute respiratory syndrome-related coronavirus
STAT3: Signal transducer and activator of transcription 3
TNF: Tumour necrosis factor
TRAF2: TNF receptor associated factor 2
UPR^ER^: ER-unfolded protein response
VSV: Vesicular stomatitis virus
Xbp1s: X-box binding protein 1 spliced
Xbp1u: X-box binding protein 1 unspliced

## INTRODUCTION

An immense number of DNA and RNA viruses from bacteria and eukaryotes populate the globe, yet, most of them are harmless to humans, because they are not adapted to vertebrate cells. Several dozens of distinct viruses nevertheless enter humans, for example, through the eyes, the skin or the respiratory, gastro-intestinal and sexual tracts (Virgin, Wherry and Ahmed 2009). Such viruses cause infectious diseases, sometimes with global impact, and emerge unpredictably.

When viruses interact with cells, they perturb homeostasis, which results in their inactivation or in acute infection (Gulbahce *et al*. 2012; Rozenblatt-Rosen *et al*. 2012; Greber 2016; Greber and Flatt 2019). A number of evolutionary conserved mechanisms guard against infections. At the organismic level, anatomical barriers, such as polarized epithelial cells and mucosal secretion protect against airborne-viruses (Holt *et al*. 2008). Defense at the tissue level is coordinated by mucosal immunity and homing of immune cells, such as macrophages, dendritic cells, regulatory T cells, helper T cells, natural killer cells and mast cells equipped with specialized sensor proteins, including scavenger receptors and toll-like receptors (Takeda and Akira 2005; Fejer *et al*. 2008; Jost and Altfeld 2013; Byrne *et al*. 2015; Maler *et al*. 2017; Schmidt and Varga 2018; Stichling *et al*. 2018; Wang *et al*. 2018; Marshall, Portales-Cervantes and Leong 2019). At the cellular level, distinct biochemical processes antagonize infections and restore homeostasis. They include the DNA damage response, glycolysis, fatty acid synthesis, oxidative stress response, heat shock response, autophagy as well as the UPR^ER^ in the endoplasmic reticulum (ER) or processes in mitochondria (for reviews, see Kudchodkar and Levine 2009; Takeuchi and Akira 2009; Haynes and Ron 2010; Heaton and Randall 2011; Chan 2014; Roulin *et al*. 2014; Sanchez and Lagunoff 2015; Chatel-Chaix *et al*. 2016; Paul and Munz 2016; Khomich *et al*. 2018; Lotzerich *et al*. 2018; Weitzman and Fradet-Turcotte 2018; Hur 2019).

Acute infections arise when viral genomes replicate, and disseminate locally and systemically. Acute tissue damage is exacerbated by pathogen-associated molecular patterns (PAMPs) of viral proteins and nucleic acids triggering pattern recognition receptors (PRRs), and an immune response through the production of interferon (IFN) and proinflammatory cytokines (Haller, Kochs and Weber 2006; Rouse and Sehrawat 2010; Hoffmann, Schneider and Rice 2015).

Viruses have evolved to antagonize the inflammatory and IFN responses, and eventually restore homeostasis in a series of complex processes crucial for both the virus and the infected organism. It is notable that the failure to attenuate inflammation and IFN signalling can lead to the death of the organism, as exemplified with SARS-CoV-2, which blunts the production of IFN in the infected cells, but leaves the inflammatory response largely unaffected, a situation which results in a cytokine storm and fatal organ failure (Blanco-Melo *et al*. 2020). In most cases, the restoration of homeostasis involves a combined action of intrinsic, innate and adaptive immunity, and comprises microRNAs, pattern recognition receptors, antibodies and cell-based immunity (Takeuchi and Akira 2009; O'Connell *et al*. 2010; Pulendran, Li and Nakaya 2010). Examples of intrinsic factors are the tripartite interaction motif 5 splice variant α (TRIM5α) blocking HIV capsid uncoating, and adenosine deaminase ADAR1 balancing immune activation and self-tolerance (Colomer-Lluch *et al*. 2018; Lamers, van den Hoogen and Haagmans 2019). Restoration of homeostasis either clears the infection, or leads to virus persistence without obvious signs of disease and immune reactions (Virgin, Wherry and Ahmed 2009).

Here, we explore how viruses use the UPR^ER^ to restore homeostasis and virus output. For detailed reviews on the UPR^ER^ in herpesvirus and coronavirus infection, we refer the reader to recent overviews elsewhere (Fung and Liu 2019; Johnston and McCormick 2019).

## THE UPR^ER^ STRESS SENSORS AND DOWNSTREAM SIGNALS

The ER has multiple functions, including the synthesis of proteins, oligosaccharides and lipids (Helenius and Aebi 2001; Ellgaard and Helenius 2003; Metcalf *et al*. 2020). Its lumen contains a high concentration of Ca^2+^ ions, and serves as both source and sink in Ca^2+^ signalling. The ER lumen is an oxidative environment and facilitates the formation of disulfide bonds in proteins, which is critical for the proper folding of newly synthesized proteins, together with protein- and lipid-glycosylation and molecular chaperones, such as the binding immunoglobulin protein (BiP, or glucose regulated protein 78, Grp78) (Xu *et al*. 2005). Newly synthesized secretory and membrane spanning proteins are properly folded in the ER, transported to intracellular organelles or secreted to the plasma membrane (Barlowe and Miller 2013). They undergo a range of modifications, including proteolytic processing, glycosylation and lipidation, interact with chaperones, isomerases, glycosyltransferases and glycosidases, and eventually exiting the ER after proper folding (Hammond and Helenius 1995; Wei *et al*. 2006; Braakman and Hebert 2013). In addition, the ER is a major hub for the synthesis of membrane lipids (Futerman and Riezman 2005; Maxfield and van Meer 2010; Harayama and Riezman 2018). The environment of the ER can be stressed by both physiological and pathological processes (Metcalf *et al*. 2020). Disturbances include the deregulation of cellular redox or the ER lipid environment, aberrant Ca^2+^ levels, glucose deprivation, or the accumulation of unfolded proteins in the ER.

ER-stress triggers an evolutionarily conserved response, the UPR^ER^. UPR^ER^ is distinct from UPR in mitochondria, which is triggered by proteotoxic signals from reactive oxygen species and exacerbated by a decrease in mitochondrial membrane potential (Rolland *et al*. 2019). The UPR^ER^ was originally found to balance the synthesis, folding and degradation of proteins in the ER (reviewed in Ron and Walter 2007; Walter and Ron 2011). When the protein load in the ER exceeds the folding capacity, or when ER homeostasis is disturbed by ectopic cues, a set of phylogenetically conserved pathways transmits signals to relieve the condition of a stressed ER (Grootjans *et al*. 2016). The sensing of ER stress occurs by transmembrane stress transducers, the inositol-requiring enzyme 1 (Ire1), the protein kinase R (PKR)-like ER kinase (PERK), and the activating transcription factor 6 (ATF6). They all sense the levels of unfolded proteins by virtue of their lumenal domains in the ER, and transmit the information through their respective cytoplasmic domains to cytosolic effector pathways (Bernales, McDonald and Walter 2006). Remarkably, the Ire1 isoform alpha (Ire1α) and PERK also sense stress from saturated lipids in the ER membrane, and transduce a remedial response through their transmembrane domain (Volmer, van der Ploeg and Ron 2013; Kono, Amin-Wetzel and Ron 2017; Metcalf *et al*. 2020).

### Ire1

Ire1 is the sensor of the most conserved branch of the UPR^ER^. It is present in lower and higher eukaryotes. In mammals, two forms of Ire1, α and β are encoded by two separate genes ERN1 and ERN2, respectively. Ire1α is expressed ubiquitously while Ire1β is primarily expressed in gastrointestinal and respiratory tracts (Bertolotti *et al*. 2000; Tsuru *et al*. 2013). Both isoforms are type-I transmembrane proteins with an N-terminal lumenal domain and a dual-function cytoplasmic domain with Ser/Thr kinase and a ribonuclease (RNase) activities (Li *et al*. 2010). Ire1 has been extensively studied in yeast. However, yeast Ire1 (yIre1) is structurally different from the human Ire1 (hIre1) α (Gardner and Walter 2011). Upon accumulation of unfolded proteins in the ER, Ire1 trans-autophosphorylates and oligomerizes. This induces conformational changes in the RNase domain, which then cleaves a small intron of the transcription factor X-box binding protein (Xbp) 1 mRNA (Xbp1u), followed by ligation yielding a spliced mRNA encoding the active transcription factor Xbp1s (Aragon *et al*. 2009; Li *et al*. 2010; Jurkin *et al*. 2014). The role of Xbp1s in UPR^ER^ homeostasis is crucial. Along with other UPR^ER^-induced transcription factors, Xbp1s upregulates and transactivates a repertoire of genes necessary for relieving the ER stress (Reimold *et al*. 2001; Acosta-Alvear *et al*. 2007). In addition, Xbp1s functions in cell growth, differentiation, survival and plasma cell differentiation, and immune cell development (Grootjans *et al*. 2016).

Although hIre1 is a sensor for protein stress in the ER, it has structurally unfavorable features for the direct binding of unfolded proteins. Hence, the question how Ire1α senses protein stress in the ER lumen, and transduces this information to the cytosol has been the subject of intense research over many years. Two main models exist for how the lumenal domain of Ire1α oligomerises and leads to the activation of the RNase function. The first model suggests that similar to yeast, unfolded proteins can bind to the core lumenal domain causing allosteric changes leading to Ire1α oligomerization (Karagoz *et al*. 2017). The unfolded proteins bind to an MHC-like groove of yIre1, whereas in hIre1, the helices flanking this groove are too closely placed to allow binding of unfolded proteins (Zhou *et al*. 2006). Dimerization and oligomerisation interfaces are separate in yIre1, whereas in hIre1, the oligomerisation interface is sterically hidden by other lumenal domains. In addition, the corresponding interface is postulated to be energetically unfavourable for oligomer formation. Yet, it is in a dynamic equilibrium between a closed- and an open-loop configuration. The open-loop structure of hIre1 can be bound and stabilised by unfolded proteins, and through allosteric changes this leads to exposure of an oligomerization interface, which promotes the formation of higher-order oligomers (Karagoz *et al*. 2017).

The second model suggests that Ire1 signalling is suppressed by the ER-resident BiP, which binds to Ire1 monomers, thereby preventing Ire1 dimerization and oligomerization (Bertolotti *et al*. 2000; Carrara *et al*. 2015). Unfolded proteins in turn also bind to BiP, and shift the equilibrium towards BiP-less Ire1 favouring Ire1 dimer and oligomer formation. Supporting this model, the co-chaperone ER-localised J-protein 4 (ERdj4) was recently shown to energetically promote the binding of BiP to Ire1α, and disrupt Ire1α dimers (Amin-Wetzel *et al*. 2017). It may be unlikely, however, that BiP dissociation from Ire1 provides the sole cue to Ire1 activation. A mutational study of the yIre1 lumenal domain removing the lumenal BiP binding site juxta membrane gave rise to Ire1, which remained inactive in the absence of ER stress, yet retained its stress-induced activation (Kimata *et al*. 2004). This opens the possibility that lumenal proteins, for example of viral origin, have evolved to directly activate Ire1α without affecting the other ER stress sensors PERK and ATF6.

Ire1 signalling has distinct downstream effects, most prominently Xbp1s, which activates a group of UPR^ER^ target genes (Acosta-Alvear *et al*. 2007). In parallel, specific degradation of a subset of ER-localized mRNAs has been identified and dubbed ‘regulated Ire1-dependent decay’ (RIDD) (Hollien and Weissman 2006). RIDD of ER-bound mRNAs may reduce the protein influx into the ER during ER stress. Exactly how Ire1 switches between Xbp1 splicing and RIDD is not clear, although weak activation of Ire1 gives rise to either Xbp1 splicing or RIDD. This opens a possibility for cytoplasmic viral regulators to toggle-switch between Xbp1 activation and mRNA decay.

In addition to RIDD, the cytoplasmic domain of phosphorylated Ire1α can interact with tumor necrosis factor (TNF)-receptor-associated factor (TRAF) 2, an adaptor protein coupling plasma membrane receptors to c-JUN N-terminal kinase (JNK) activation and pro-apoptotic stimulation (Urano *et al*. 2000). A recently discovered process by which terminally misfolded-proteins are degraded in the ER by a process termed ‘ER-associated degradation’ (ERAD) (reviewed in Smith, Ploegh and Weissman 2011). ERAD contributes to ER homeostasis by removing terminally misfolded proteins from the ER and targeting them for proteasomal degradation. Protein extraction from the ER involves the AAA+adenosine triphosphatase (ATPase) p97 (valosin-containing protein in humans) (reviewed in Metcalf *et al*. 2020). The ERAD is directly linked to the UPR^ER^, as ER stress-induced Xbp1s transcriptionally upregulates components of the retrotranslocation machinery for protein transport from the ER to the cytosol (Iwakoshi, Lee and Glimcher 2003; Acosta-Alvear *et al*. 2007; Araki and Nagata 2012).

### PERK

PERK is a type-I transmembrane protein with a lumenal stress sensing domain, and a cytoplasmic kinase domain which trans-autophosphorylates upon ER stress-induced oligomerisation. Unlike Ire1, PERK phosphorylates the alpha subunit of eukaryotic translation initiation factor 2 (eIF2α), and thereby suppresses translation initiation (Harding, Zhang and Ron 1999). PERK-mediated eIF2α phosphorylation inhibits the guanine-nucleotide exchange factor eIF2B. eIF2B accelerates the exchange of GDP for GTP in the eukaryotic initiation factor 2 (eIF2) complex (Ranu and London 1979). Although activation of PERK leads to a strong suppression of protein synthesis, some mRNAs with short inhibitory open reading frames (uORFs) in the 5’ untranslated region (5’UTR) resist translation inhibition by PERK. For example, the UPR^ER^ transcription factor ATF4 is expressed preferentially upon PERK activation (Lu *et al*. 2004; Vattem and Wek 2004). ATF4 enhances the pro-apoptotic C/EBP homologous protein (CHOP), which downregulates the anti-apoptotic protein Bcl-2 (B-cell lymphoma 2) and promotes cell death via cytochrome c release (Harding *et al*. 2000; Ma *et al*. 2002).

It is worth noting that ribosomal subunits are post-translationally modified in uninfected cells. For example, the induction of the UPR^ER^ induces site-specific ubiquitination on RPS3, RPS2 and RPS20 with possible functional consequences for translation (Higgins *et al*. 2015). Apart from a stressed ER, starvation or heme depletion can lead to phosphorylation of eIF2α. Additionally, protein kinase (PKR) is a cytoplasmic type-I IFN-induced enzyme, which can also phosphorylate eIF2α and enhance apoptosis by upregulating ATF4 and CHOP. Hence, this signalling arm is commonly referred to as the integrated stress response (ISR) (Harding *et al*. 2003). Recent findings suggest that ISR is persistently active in mice with traumatic brain injury leading to continuous eIF2α phosphorylation (Chou *et al*. 2017). The small-molecule drug-like compound ISRIB (ISR inhibitor) promotes eIF2B dimerization causing enhanced activity on its substrate eIF2 independent of upstream inhibition of eIF2α (Sekine *et al*. 2015; Sidrauski *et al*. 2015; Tsai *et al*. 2018; Zyryanova *et al*. 2018). Hence, ISRIB blunts the block on translation initiation, and was shown to enhance cognition in rodents with traumatic brain injury.

### ATF6

ATF6 represents a third type of transmembrane ER stress sensor. When unfolded proteins accumulate in the ER lumen, ATF6 is transported to the Golgi apparatus in COPII vesicles (Schindler and Schekman 2009). At the Golgi, ATF6 gets processed by two proteases, S1P (site-1) and S2P (site-2) proteases, which remove the lumenal and transmembrane anchor, respectively (Haze *et al*. 1999; Ye *et al*. 2000). This gives rise to N-terminal ATF6 (ATF6-N), which acts as a potent UPR^ER^ transcription factor. In the nucleus, ATF6-N enhances UPR^ER^ gene transcription by binding to cis-acting ER stress response elements (ERSE). Prominent target genes include Xbp1 and BiP/Grp78 (Yoshida *et al*. 2000). ATF6-N also enhances CHOP mRNA levels and promotes pro-apoptotic signals in the terminal phase of the UPR^ER^ (Yoshida *et al*. 2000), together with the activating transcription factor 4 (ATF4), which is enhanced by PERK.

## VIRUSES ACTIVATE AND SUPPRESS THE UPR^ER^

**Figure 1.**
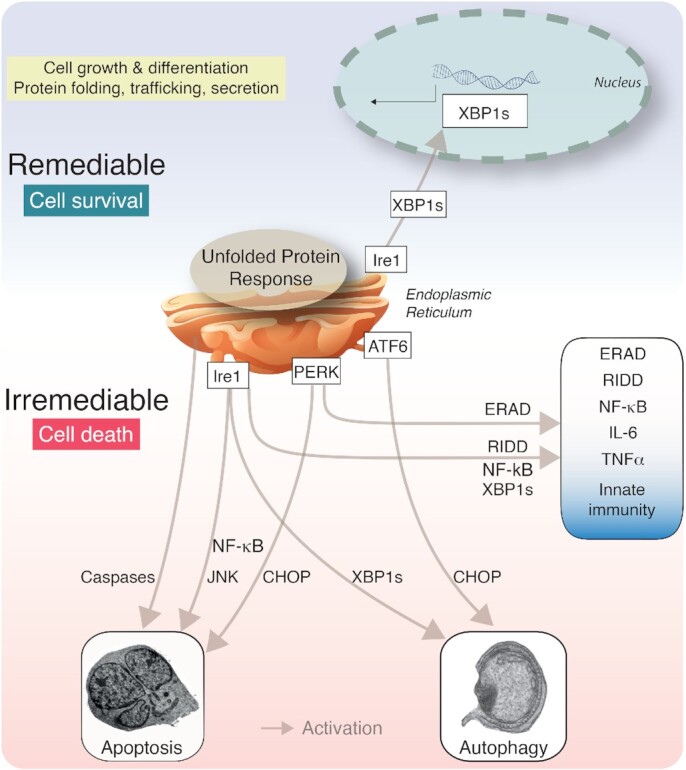
The major signalling channels of the UPR^ER^ in cell survival, death and innate immunity. Distinct signalling pathways downstream of the vertebrate UPR^ER^ sensors result in cell survival, death and innate immunity. Abbreviations: Ire1, Inositol-requiring enzyme 1; Xbp1s, X-box binding protein 1 spliced; ATF6, Activating transcription factor 6; PERK, Protein kinase activated by double stranded RNA (PKR)-like ER kinase; NF-κB, Nuclear factor kappa light-chain-enhancer of activated B cells; JNK, c-Jun N-terminal kinases; CHOP, CCAAT/-enhancer-binding protein homologous protein; RIDD, Regulated Ire1-dependent decay of mRNA; ERAD, ER-associated degradation; IL-6, Interleukin-6; TNFα, Tumour necrosis factor alpha.

The UPR^ER^ maintains homeostasis by multiple effector pathways, including a transcriptional upregulation of protein-folding enzymes (so-called chaperones), enhancement of the ERAD, and reduction of global protein synthesis. The type and duration of stress are crucial for the overall output of the UPR^ER^. Under relatively mild ER stress, the Ire1-Xbp1s arm can remedy the detrimental effects of unfolded protein accumulation. The Ire1α activated transcription factor Xbp1s binds and enhances the expression of a subset of genes promoting cell survival, growth and differentiation, including protein biosynthesis and folding, trafficking and secretion (Acosta-Alvear *et al*. 2007). See Fig. 1.

**Figure 2.**
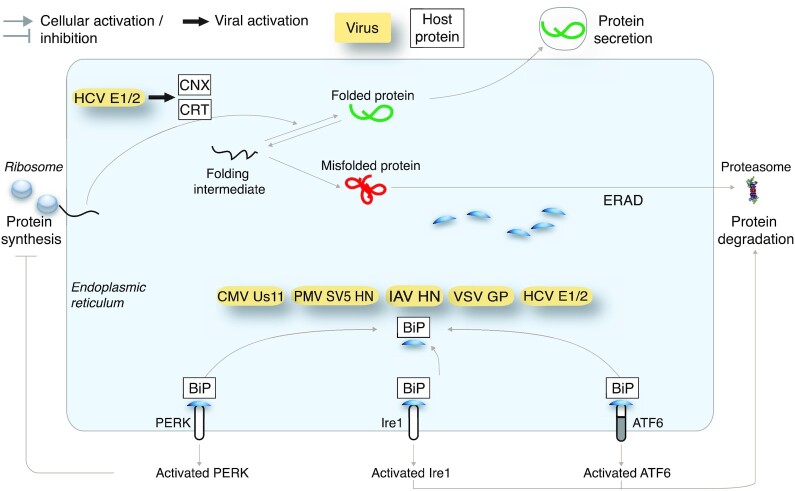
Viruses sequestering BiP/Grp78 in the ER lumen. Initiation of a global UPR^ER^ in virus infections can occur by the sequestration of the ER chaperone BiP/Grp78 from the lumenal domain of the UPR^ER^ sensor proteins Ire1α, PERK and ATF6. All signalling arms of the UPR^ER^ sensors are activated as a result of BiP/Grp78 removal from ER lumenal domains of the sensor. Abbreviations: BiP, Binding immunoglobulin protein; CNX, Calnexin; CRT, Calreticulin; HCV, Hepatitis C virus; PMV, Paramyxo simian virus; IAV, Influenza A virus; CMV, Cytomegalovirus; VSV, Vesicular stomatitis virus; ERAD, ER-associated segradation; Ire1, Inositol-requiring enzyme 1; PERK, Protein kinase activated by double stranded RNA (PKR)-like ER kinase; ATF6, Activating transcription factor 6.

**Figure 3.**
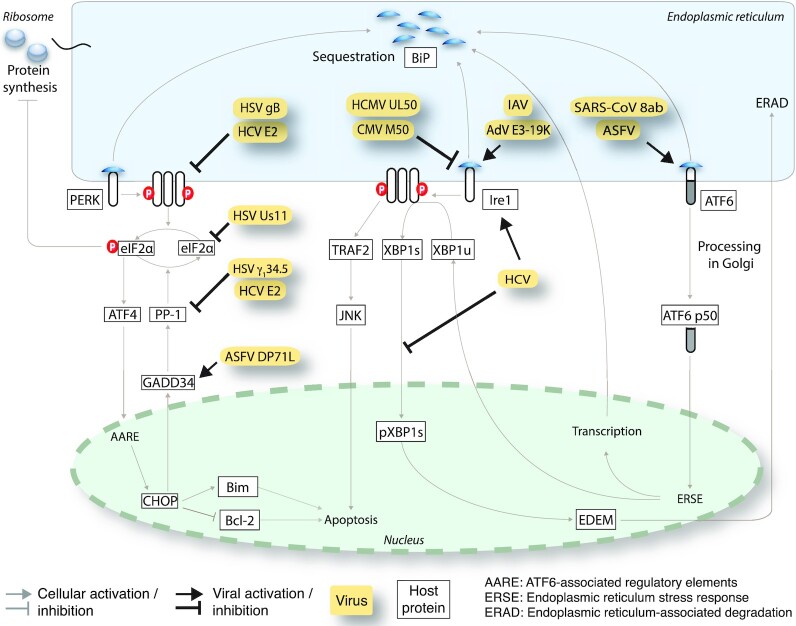
Viral proteins interfering with signal transduction along the three UPR^ER^ branches Ire1, PERK and ATF6. Examples of viruses and viral proteins that activate or inactivate specific arm of the UPR^ER^ signalling by direct or indirect interactions at the level of the sensors or downstream signal transducers. Abbreviations: BiP, Binding immunoglobulin protein; eIF2α, Eukaryotic translation initiation factor 2 subunit1; Ire1, Inositol-requiring enzyme 1; ATF6, Activating transcription factor 6; PERK, Protein kinase activated by double stranded RNA (PKR)-like ER kinase; HSV, Herpes simplex virus; HCV, Hepatitis C virus; HCMV, Human cytomegalovirus; CMV, cytomegalovirus; IAV, Influenza A virus; AdV, Adenovirus; ASFV, African swine fever virus; ATF4, Activating transcription factor 4; PP-1, Protein phosphatase 1; CHOP, CCAAT/-enhancer-binding protein homologous protein; BiM, Bcl-2 like protein 11; Bcl-2, B-cell lymphoma 2; AARE, Amino acid response element; EDEM, ERAD enhancing α-mannosidase-like proteins; Xbp1s, X-box binding protein 1 spliced; JNK, c-Jun N-terminal kinases; TRAF2, TNF receptor associated factor 2.

Enveloped viruses commonly require large amounts of properly folded membrane glycoproteins leading to ER overload, and activation of a global UPR^ER^ involving all three sensors Ire1α, PERK and ATF6. Examples of viral proteins that bind and sequester the ER chaperone BiP/Grp78 away from the lumenal domain of Ire1α, PERK and ATF6 are listed in Fig. 2. The induction of a strong UPR^ER^ may blunt infection, for example, by attenuation of translation through PERK activation, triggering premature apoptotic cell death or immune responses (Walter and Ron 2011; Smith 2014). To balance UPR^ER^ signalling viruses have evolved strategies to either activate or inhibit particular arms of the UPR^ER^, as depicted in Fig. 3. Below, we provide a discussion of select viruses that activate or suppress the UPR^ER^.

## VIRAL ACTIVATION OF THE UPR^ER^

The following nine enveloped viruses (in alphabetical order) induce the UPR^ER^ by ER overload or specific signals from the ER lumen.

***African swine fever virus (ASFV)***—ASFV infection enhanced the expression of ER chaperones, particularly calnexin and calreticulin (Galindo *et al*. 2012). This increase has been linked to the activation of ATF6 but a specific role of viral proteins is unknown.

***Cytomegalovirus (CMV)***—The CMV Us11 protein is sufficient to induce the UPR^ER^ as suggested by the upregulation of BiP levels and Xbp1 splicing (Tirosh *et al*. 2005). See Fig. 2. Recent studies showed that murine CMV (MCMV) early gene expression is suppressed by the unspliced form of the Xbp1 mRNA (Xbp1u) (Hinte *et al*. 2020). MCMV transiently activated the Ire1α-Xbp1 axis, depleted Xbp1u and relieved the transcriptional repression of the immediate early viral promoter boosting viral replication. The study also showed an unexpected role of Xbp1u as a potent repressor of both XBP1s and ATF6-mediated activation.

***Dengue virus (DENV)***—A generalised activation of UPR^ER^ pathways has been reported in DENV infection (Umareddy *et al*. 2007; Datan *et al*. 2016; Perera, Miller and Zitzmann 2017), without much information on viral proteins involved, although an induction of Xbp1s was reported (Yu *et al*. 2006).

***Hepatitis C virus (HCV)***—HCV encodes two envelope glycoproteins E1 and E2, which form non-covalent heterodimers and higher order oligomers. These glycoproteins bound to ER chaperones, including BiP, calnexin and calreticulin (Choukhi *et al*. 1998). The expression of E2 led to a generalised activation of all the UPR^ER^ sensors, and increased Xbp1s (Tardif *et al*. 2004; Chusri *et al*. 2016), phosphorylated PERK, cleaved ATF6 (Chusri *et al*. 2016), and an increase in BiP transcription (Liberman *et al*. 1999). See Fig. 2.

***Influenza* *A* *(IAV)***—Initially, misfolded hemagglutinin precursor protein HA0 was found to non-covalently associate with BiP (Hurtley *et al*. 1989) (Fig. 2). Later studies showed that IAV infection mainly activated the Ire1α pathway, whereas PERK and ATF6 activities were either unaffected or suppressed (Hassan *et al*. 2012). The stabilization of the UPR^ER^ by the bile component tauroursodeoxycholic acid (TUDCA) reduced Ire1α activation, activated PERK and phosphorylated eIF2α, decreased protein synthesis, and promoted the expression of ATF4 (Hassan *et al*. 2012; Kusaczuk 2019). Intriguingly, TUDCA suppressed IAV titers, possibly by unbalancing the UPR^ER^ network to maintain homeostasis. Alternatively, the ER stress-induced innate and adaptive immune responses, including NF-κB signalling (described in detail in section 4 and 6) and the type I IFN response may be harnessed in IAV infection to tune cell survival and virus output (Liu *et al*. 2012; So 2018).

***Japanese encephalitis virus (JeV)***—JeV belongs to the genus *Flavivirus*, and its infection induces all branches of the UPR^ER^, as seen directly by PERK phosphorylation, Xbp1 splicing and ATF6 cleavage (Yu *et al*. 2006; Sharma *et al*. 2017).

***Paramyxo simian virus 5 (PMV SV5)***—Both the unfolded and folded forms of the viral hemagglutinin-neuraminidase protein were shown to form a complex with BiP and might be causing an induction of a broad UPR^ER^ (Ng *et al*. 1989). A subsequent study reported an increase in the transcription of UPR^ER^ genes with ectopic HN expression and in SV5 infection (Watowich, Morimoto and Lamb 1991). See Fig. 2.

***SARS Coronavirus (SARS-CoV)***—The 8ab protein of SARS-CoV, which locates to the lumen of the ER, induces ER-resident chaperones and ATF6 activation, apparently without PERK or IRE1α activation, as evidenced by absence of CHOP induction or and Xbp1 splicing, respectively (Sung *et al*. 2009) (Fig. 2).

The expression of 8ab led to ATF6 cleavage and promoted the nuclear translocation of its transcription-active amino terminal domain ATF6-N. A subsequent study showed that an 18-amino acid long peptide of 8ab interacted with the Ire1α lumenal domain *in vitro* (Karagoz *et al*. 2017). Whether this interaction leads to activation of Ire1α in cells has remained unknown. Another study reported that SARS-CoV spike protein enhanced BiP/Grp78 expression via PERK pathway of UPR^ER^ (Chan *et al*. 2006).

***Vesicular stomatitis virus (VSV)***—A specific population of VSV G-protein that formed incomplete disulphide bonds and transiently interacted with BiP/Grp78, albeit without specific information on the UPR^ER^ induction (Machamer *et al*. 1990). Later studies showed eIF2a phosphorylation as an indicator of PERK activation, and other downstream UPR^ER^ genes (Connor and Lyles 2005; Liu *et al*. 2009). See Fig. 2.

The following three nonenveloped viruses induce the UPR^ER^ by expressing viral nonstructural proteins in the ER.

***Adenovirus (AdV)***—Initially, AdV infection was found to be increased by small chemical compounds enhancing the UPR^ER^, such as Golgicide A, or RNA interfence against genes controlling ER-Golgi trafficking (Prasad *et al*. 2014). The enhancing effects on the early viral gene expression were dependent on the Ire1α-Xbp1 axis of the UPR^ER^. Subsequent studies showed that AdV infection also enhanced the UPR^ER^. In particular, the ER lumenal domain of the viral glycoprotein E3-19K formed a complex and specifically activated the Ire1α branch of the UPR^ER^ for extended periods (Prasad *et al*. 2020). The activation of Ire1α in the context of infection or upon expression of ER-directed E3-19K lumenal domain alone enhanced the splicing of Xbp1u to Xbp1s mRNA. In the infected cells, E3-19K promoted early viral gene expression through Xbp1s binding to the E1 and the E4 promoters, as demonstrated by chromatin immunoprecipitation and E1 promoter mutagenesis. Ire1α activation by E3-19K occurred noncanonically, that is, without increase of BiP/Grp78, RIDD or PERK and ATF6 activations. In addition, pre-existing BiP dissociated from Ire1α before Ire1α activation measured by XBP1 splicing was observed. The extended Ire1α activation promoted the long term persistence of AdV in cell cultures in the presence of IFN. AdV mutants lacking E3-19K and pharmacological interference with the Ire1α nuclease activity abrogated persistence and virus disappearance from the cultures (Cross *et al*. 2012; Prasad *et al*. 2020).

***Adeno-associated virus (AAV)***—Transductions of cultured cells with recombinant self-complementary AAV1 and AAV6 (or AAV2 in hepatic transductions) were shown to induce Ire1α and PERK mRNA levels, and AAV6 also induced ATF6 mRNA suggesting viral capsid dependent effects on the UPR^ER^ sensors (Balakrishnan *et al*. 2013). RNA interference-mediated inhibition of Ire1α and PERK, however, gave only minimal effects on AAV2 and AAV6 transduction, suggesting that the UPR^ER^ sensor induction was not a proviral response. Whether a pharmacological inhibition of the UPR^ER^ can be applied in combination with AAV transduction in clinical settings remains an open question.

***Coxsackievirus (CV)***—Infection with CVB3 induced the canonical UPR^ER^, with increased BiP levels, activated Ire1α and PERK, increased ATF6-N, and enhanced expression of UPR^ER^ target genes (Zhang *et al*. 2010). However, interactions and direct actions of stressors on the UPR^ER^ sensors and regulators have remained unknown.

## VIRAL SUPPRESSION OF THE UPR^ER^

The production of progeny in infected cells requires the synthesis of viral structural proteins in excess over those that are actually incorporated into the particles. This is because low affinity and high avidity cooperative interactions between virion proteins themselves and the viral genome control the assembly of the particles. Low affinity/high avidity assembly gives rise to infectious particles which are able to respond to host cues for uncoating in naïve cells (reviewed in Yamauchi and Greber 2016; Greber 2019; Greber and Flatt 2019). Interestingly, virus-like particles can be evolved to package RNA synthetically, but they remain unresponsive to the uncoating cues in the target cells, as for example indicated by recent laboratory evolution of the bacterial enzyme lumazine synthase from Aquifex bacteria (Tetter *et al*. 2020). This highlights the importance of combinatorial evolutionary selection processes in virus biogenesis, and puts an exciting perspective for the UPR^ER^ in synthetic biology, considering the possibility of evolvable protein cages with tunable assembly and disassembly functionality (Malay *et al*. 2019). Viruses in nature have solved the issue by expressing an excess of virion proteins, a situation that leads to protein overload in the ER and elicits a canonical UPR^ER^ with anti-viral responses, as listed above. Both enveloped and non-enveloped viruses have evolved mechanisms to modulate the anti-viral facettes of the UPR^ER^, as discussed below (see also Fig. 3).

Here we list five enveloped and nonenveloped viruses, which circumvent or suppress aspects of the UPR^ER^ in the course of progeny formation.

***Adenovirus (AdV)***—As discussed above, the canonical UPR^ER^ induction and PERK activation are absent in AdV infections (Prasad *et al*. 2020). Nonetheless, AdV activates double-stranded RNA activated protein kinase (PKR). PKR is a cytoplasmic type-I IFN-induced enzyme, which phosporylates eIF2α and enhances apoptosis by upregulating ATF4 and CHOP (Lee *et al*. 2007). Since eIF2α can be phosphorylated by both PERK and PKR, translation inhibition in AdV infection occurs even without PERK activation. The virus uses several strategies to block translation inhibition by phosphorylated eIF2α. Late in infection, global translation shutoff is prevented by viral associated RNA I (VA RNA I), which is a PKR inhibitor and acts as a decoy of double-stranded RNA which normally activates PKR (Mathews and Shenk 1991). In addition, AdV E1B-55K and E4orf6 proteins form a ubiquitin ligase complex, which inhibits the phosphorylation of eIF2α (Harada *et al*. 2002; Spurgeon and Ornelles 2009).

***African swine fever virus (ASFV)***—During ASFV infection, inhibition of CHOP expression was reported (Netherton, Parsley and Wileman 2004). The ectopically expressed ASFV protein DP71L, a homolog of protein phosphatase 1 regulatory subunit 15A, interacted with protein phosphatase 1 catalytic subunit (PP1c), recruited PP1c to eIF2α and led to eIF2α dephosphorylation (Zhang *et al*. 2010). The importance of eIF2α dephosphorylation was further emphasized by the finding that mutant viruses lacking DP71L still reduced eIF2α phosphorylation and CHOP levels, suggesting that virus has redundant mechanisms for eIF2α dephosphorylation (Fig. 3).

***Cytomegalovirus (CMV)***—Not a lot is known about PERK and eIF2α suppression in CMV infection, but the Ire1-Xbp1 arm of the UPR^ER^ was suppressed by the murine CMV M50 protein. The N-terminal region of M50 was shown to be present in a complex with Ire1α leading to a decrease in Ire1α levels via an unknown degradation pathway (Stahl *et al*. 2013). Similar observations were made with UL50, the human homolog of ML50. We speculate that the reduction of Ire1α levels in CMV infections is key to prevent an overreaction of the UPR^ER^, when the viral protein load in the ER increases for virion morphogenesis.

***Herpes simplex virus (HSV)***—Several strategies have been reported by which HSV circumvents the global translational shutdown downstream of an activated PERK branch. For example, the ICP0 promoter of HSV1 responded to ER stress, and speculatively, may tune the UPR^ER^ early in infection (Su *et al*. 2016). In addition, the viral glycoprotein gB binds to PERK and reduces its phosphorylation (Mulvey, Arias and Mohr 2007). Further, two viral proteins (Us11 and ϒ_1_34.5) targeted eIF2α phosphorylation to prevent a global translation shutdown upon PERK activation (Cheng, Feng and He 2005). HSV ϒ_1_34.5 was expressed before viral replication and interacted with cellular phosphatase PP1a to cause eIF2α dephosphorylation (Chou *et al*. 1995; Mulvey *et al*. 2003) (Fig. 3). The ϒ_1_34.5 gene may provide an example of evolutionary mimicry, since it has a C-terminal domain homologous to GADD34, an activator of PP1a which dephosphorylates eIF2a and prevents host cell protein synthesis shutoff (Chou and Roizman 1994; He *et al*. 1997).

These examples illustrate apparently redundant HSV strategies for effectively maintaining translation in the background of an ongoing UPR^ER^. In addition to the viral modulation of PERK activity, the ectopic expression of the viral protein UL41 has been shown to suppress Ire1α RNase function and Xbp1 splicing promoted by the ER stress inducer thapsigargin, which inhibits the Ca^+2^ ATPase in the ER and depletes Ca^+2^ stores in the ER (Zhang *et al*. 2017). This interference may effectively blunt an overreacting Ire1α response and preserve homeostasis in the infected cell. For recent overviews on the UPR in herpesvirus and porcine reproductive and respiratory syndrome virus infections, see (Gao *et al*. 2019; Johnston and McCormick 2019).

**Figure 4.**
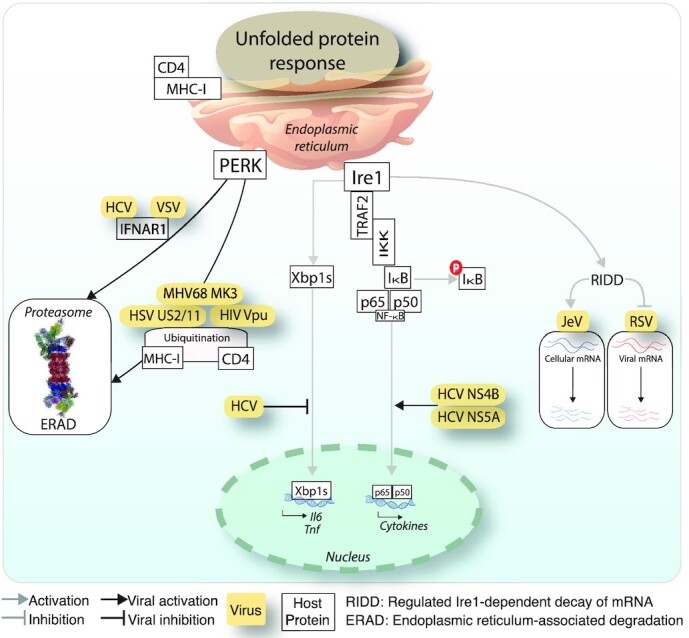
Viral modulation of innate immunity effectors interlinked with the Ire1 and PERK branches of the UPR^ER^. Viruses intercept signalling from the Ire1-Xbp1 axis to innate immunity hubs by interfering with activated NF-κB or RIDD. This can trigger proviral effects by degradation of host mRNAs or raise susceptibility to antiviral effects, for example when RIDD degrades viral RNA. PERK signalling can be activated by viruses to target key innate immunity components, such as IFNAR1 or MHC-I for proteasomal degradation. Abbreviations: PERK, Protein kinase activated by double stranded RNA (PKR)-like ER kinase; Ire1, Inositol-requiring enzyme 1; TRAF2, TNF receptor associated factor 2; IKK, IκB kinase; CD4, Cluster of differentiation 4 glycoprotein; MHC-I, Major histocombatibility factor I; RIDD, Regulated Ire1-dependent decay of mRNA; JeV, Japanese encephalitis virus; RSV, Respiratory syncytial virus; ERAD, ER-associated degradation; IL-6, Interleukin-6; TNF, Tumour necrosis factor; HIV, Human immunodeficiency virus; HSV, Herpes Simplex Virus; MHV, Mouse gammaherpes virus; HCV, Hepatitis C virus; VSV, Vesicular stomatitis virus; IFNAR1, Interferon alpha or beta receptor subunit 1.

***Hepatitis C virus (HCV)***—Although the HCV E1 and E2 glycoproteins led to the activation of all UPR^ER^ sensors, several reports show that the downstream signalling of the UPR^ER^ is inhibited by HCV. Specifically, HCV replicon-containing cells showed enhanced Xbp1 splicing but transcriptional enhancement of Xbp1 target genes of the host was rather modest (Tardif *et al*. 2004). This suggests that Xbp1s target genes, such as EDEM (ER degradation-enhancing alpha-mannosidase-like protein) which enhances the degradation of misfolded proteins, require additional input besides Xbp1s for robust transcription stimulation. Accordingly, Ire1α-knockout cells displayed elevated levels of HCV glycoproteins, possibly because EDEM was reduced in these cells (Tardif *et al*. 2004). It can be speculated that HCV suppresses the Ire1-Xbp1 axis to promote viral envelope protein expression. An additional beneficial effect for the suppression of Ire1-Xbp1 by HCV might be to reduce the activity of transcription factors, which normally increase the pro-inflammatory cytokine production in response to viral pattern recognition receptor engagement (Smith 2014) (Fig. 4). A possible evolutionary mimicry for the shut down of UPR^ER^ has also been described for HCV infection. The HCV E2 protein contains a sequence identical to the phosphorylation sites of IFN-inducible PKR and its target eIF2α. By this interaction, E2 binds and inhibits activity of PKR and neutralizes its inhibitory effect on protein synthesis, thereby contributing to the IFN resistance of HCV infections (Taylor *et al*. 1999).

## THE UPR^ER^ IN INNATE IMMUNITY

The immune response is key to the defense of cells and organisms against pathogens, and can severely affect the ER, and the restoration of homeostasis. ER stress and the UPR^ER^ play emerging roles in immunity and exacerbate the cell sensitivity to inflammatory stimuli (Bettigole and Glimcher 2015). The UPR^ER^ shares several signalling modules with immune response pathways (see Fig. 1). For example, increase in NF-κB transcriptional activity by PERK and Ire1α is linked to pro-inflammatory cytokine production in macrophages through NF-κB signalling. This happens through increased NF-κB levels compared to the short-lived NF-κB inhibitor IκB, notably in presence of translation shutdown by eIF2α phosphorylation (Deng *et al*. 2004). On the other hand, Ire1α activation of NF-κB signalling is linked to Ire1α interaction with the adaptor protein TRAF2 (Hu *et al*. 2006). Dimers of NF-κB p65 and p50 RelA subunits are in an inactive state sequestered in the cytoplasm by ankyrin-repeats of the inhibitor of κB (IκB) (reviewed in Napetschnig and Wu 2013). Interaction of Ire1α with TRAF2 recruits IκB kinase (IKK), which phosphorylates IκB and thereby releases NF-κB for nuclear translocation, and IκB for degradation (Liu *et al*. 1996; Hu *et al*. 2006). In addition, TLR antagonists enhance the recruitment of TRAF6 to Ire1α via the NADPH oxidase protein, NOX2, thereby activating Ire1α and enhancing Xbp1 splicing (Martinon *et al*. 2010). This activation of Ire1α is important for cytokine production, for example interleukin-6 (IL-6), TNF and IFNß. Innate immune signalling from TNFα further induces Ire1α activity and leads to NF-κB dependent apoptotic cell death. On the contrary, the activation of glycogen synthase kinase 3ß (GSK3ß) by Ire1α together with TNF upregulation has been implicated in the down-modulation of Xbp1 transcription, and thereby fine-tunes the inflammatory response (Kim *et al*. 2015). Further, the survival of infected macrophages is supported by TLR activation, which favours the suppression of the ATF4/CHOP signalling in the PERK branch (Metcalf *et al*. 2020).

Below, we depict major overlaps between UPR^ER^ branches and innate immune responses, and discuss examples of viral down-modulation of the corresponding elements to evade cell death and clearance by the immune system.

Innate immunity and pro-inflammatory cytokines Work with flaviviruses, such as Dengue, Zika, West Nile and Tick-borne encephalitis viruses suggested that the UPR^ER^ can accelerate innate immunity by boosting the expression of IFN-stimulated genes, such as the IFN regulatory factor (IRF) 3, and thereby enhance an antiviral response (Carletti *et al*. 2019). HCV inhibition of Xbp1 transactivation provides an example for a viral strategy of immune evasion, which might contribute to viral persistence in hepatocytes (Tardif *et al*. 2004). The mechanisms are unknown, however, and it remains to be elucidated how Xbp1 down-modulation supports immune evasion of HCV.

*NF-κB*—The HCV proteins NS5A and NS4B were described to trigger the nuclear translocation of NF-κB, induce the UPR^ER^, as well as reactive oxygen species (Gong *et al*. 2001; Li *et al*. 2009). Similarly, the AdV glycoprotein E3-19K causes NF-κB nuclear translocation (Pahl *et al*. 1996), possibly through the activation of Ire1α (Prasad *et al*. 2020). Interestingly, the AdV E3 promoter contains NF-κB but not Xbp1s binding motifs, giving rise to the possibility that NF-κB contributes to the maintenance of E3 levels, and thereby enhances immune defense and virus persistence (Williams *et al*. 1990; Lichtenstein *et al*. 2004; Prasad *et al*. 2020).

***Virus-triggered ERAD of host innate immunity proteins***—Several viruses were reported to use ERAD to degrade host components with anti-viral activity (for a review see Frabutt and Zheng 2016). For example, HCMV US2 and US11 proteins bound and dislocated MHC-I heavy chains for degradation in the cytoplasm (van der Wal, Kikkert and Wiertz 2002). Similarly, murine gammaherpes 68 (MHV68) encodes an E3-ubiquitin ligase MK3, which associated with a cellular E2 ubiquitin-conjugating enzyme and MHC-I, leading to the ubiquitylation and ERAD-mediated proteasomal degradation of MHC-I (Boname and Stevenson 2001). In HIV-1 infected cells the viral accessory protein Vpu promoted virion release and prevents superinfection by downregulating the virus receptor CD4 from the cell surface (Margottin *et al*. 1998; Magadan *et al*. 2010). Vpu interacted with CD4 in the ER and induced CD4 degradation depending on ERAD by forming an ion conductive membrane pore and retargeting of a E3-ubiquitin ligase to the ER (Strebel 2014). See Fig. 4. VSV infection degrades host proteins involved in innate immunity. A specific activation of the PERK pathway led to the proteasomal degradation of type-I IFN receptor (IFNAR) 1, reducing the anti-viral IFN signalling (Baltzis *et al*. 2004; Krishnamoorthy *et al*. 2008). This finding was further supported by experiments with PERK knockdown cells where the viral protein expression was reduced and IFN signalling restored (Fig. 4). A similar mechanism of virus-induced IFNAR1 degradation was shown with HCV infection (Liu *et al*. 2009).

***Virus-triggered degradation of host mRNA by RIDD***—ER stress can induce hyper-phoshorylation of UPR^ER^ effectors. Under these conditions, Ire1α catalyzed RIDD, non-specific cleavage of mRNAs (Hollien *et al*. 2009, Walter and Ron 2011). It remains unknown if these RNA fragments were sensed by RIG-I and thereby led to the activation of NF-κB and inflammatory cytokine production, such as IL-6 and IL-8, as suggested for bacteria (reviewed in Lencer *et al*. 2015). In JeV infected cells, RIDD degraded host RNAs without affecting viral RNAs (Bhattacharyya, Sen and Vrati 2014). Blunting Ire1-RIDD activity with chemical inhibitors reduced viral titers suggesting a pro-viral effect of RIDD. However, the molecular mechanisms by which the viral transcripts escape RIDD have remained unclear. In another study, Ire1 activation in respiratory syncytial virus (RSV) infection reduced number of viral transcripts and translation products, suggesting that RIDD degraded viral transcripts as part of an anti-viral response (Hassan *et al*. 2014). See Fig. 4.

## THE UPR^ER^ IN AUTOPHAGY

Autophagy is a conserved self-eating process to encapsulate intracellular components into autophagosomes, including organelles, soluble and aggregated proteins and foreign bodies, and content degradation in lysosomes (reviewed in Yu *et al*. 2018). The sustained activation of the Ire1α-Xbp1 axis has been implicated in triggering an autophagic response by the transcriptional enhancement of autophagy effectors Beclin-1 and LC3 (Adolph *et al*. 2013; Margariti *et al*. 2013). In addition, the UPR^ER^ controlled transcription factors ATF4, CHOP, NF-κB and STAT3 upregulate the autophagosome machinery (reviewed in Pietrocola *et al*. 2013). It is conceivable that damaged ER proteins exceeding the degradation capacity of the ERAD lead to the turnover of ER segments by autophagy clearing proteins and also lipids (Houck *et al*. 2014). This goes along with the notion that autophagy counterbalances ER expansion during the UPR^ER^ (Bernales, McDonald and Walter 2006).

Below, we discuss examples of enveloped viruses using autophagic activity in connection with the UPR^ER^.

***Coronaviruses (CoVs)***—Ire1α was found to be activated in cells infected with avian infectious bronchitis virus (IVB) and served as a survival factor by antagonizing apoptosis through modulating the phosphorylation status of proapoptotic JNK and the prosurvival through RAC-alpha serine/threonine-protein kinase (Akt) (Fung, Liao and Liu 2014). The data suggest that the UPR^ER^ constitutes a major aspect of coronavirus-host interactions.

***Hepatitis C virus (HCV)***—The HCV-induced UPR^ER^ activates autophagy, and thereby enhances viral RNA replication (Ke and Chen 2011). Enhanced RNA replication depended on autophagic protein degradation, and the UPR^ER^ might have been involved in suppressing IFNß activation and immune response to infection. Notably, the IFNß activation in response to HCV PAMPs occurred only in the absence of host UPR^ER^ or absence of autophagy components. In addition, HCV inhibited the AKT-TSC-MTORC1 pathway via ER stress, and may thereby contribute to the establishment of the HCV-induced autophagy (Huang *et al*. 2013).

***Dengue virus (DENV)***—DENV associated pathogenicity and viral load were shown to depend on the PERK-eIF2α and Ire1α-JNK signalling arms of the UPR^ER^. DENV infection induced autophagy by phosphorylating Bcl-2 via the JNK signalling pathway, and promoted the dissociation of its complex with Beclin-1, thereby freeing up Beclin-1 to assemble with pre-autophagosomes (Marquez and Xu 2012). Chemical inhibition of JNK signalling reduced virus-mediated autophagy and virus titers in mice, suggesting a pro-viral role of autophagy in DENV infection (Lee *et al*. 2018). DENV infection also led to the degradation of host innate immune components by autophagy, akin to HCV infections (Ke and Chen 2011).

***Japanese encephalopathy* *virus (JeV)***—In contrast to HCV and DENV, ER stress induction in JeV infection enhanced autophagy, and reduced the viral titers and cell death. Silencing of the Xbp1 and ATF6 branches of the UPR^ER^ led to a suppression of JeV induced autophagy, and enhanced viral pathogenicity (Sharma *et al*. 2017)

***Foot-and-mouth disease virus (FMDV)—*** Recently, FMDV-induced ER stress via PERK activation has been suggested to lead to enhanced autophagy. Knockdown of both PERK and components of the autophagy machinery reduced the viral titers and enhanced antiviral innate interferon response (Ranjitha *et al*. 2020).

## THE UPR^ER^ IN CELL DEATH

Persistent and high intensity UPR^ER^ signalling can trigger an irremediable cell death program (see Fig. 1). This has been shown, for example, with lethal doses of the ER stress-inducing small chemicals thapsigargin or tunicamycin that trigger only transient Ire1α and ATF6 activity, but long-lasting PERK activity (Lin *et al*. 2007). Ire1α triggers pro-apoptotic signalling via interaction with the adaptor protein TRAF2, resulting in the activation of c-Jun N-terminal kinases (JNK) and nuclear factor kappa enhancer of B-cell (NF-κB) signalling (Urano *et al*. 2000; Hu *et al*. 2006). Binding of TRAF2 to Ire1α specifically leads to recruitment and activation cleavage of pro-caspases on the ER membrane, such as caspase-12 in mice and caspase-4 in humans (Nakagawa and Yuan 2000; Yoneda *et al*. 2001; Rao *et al*. 2002; Hitomi *et al*. 2004; Rosati *et al*. 2010). NF-κB in turn transcriptionally regulates pro-inflammatory genes with either pro-survival or apototic effects. Besides binding of TRAF2 and NF-κB, the cytosolic domain of Ire1α also binds the pro-apoptotic proteins Bcl-2 associated X-protein (Bax) and Bcl-2 homologous antagonist or killer (Bak). This binding promotes the splicing of Xbp1 mRNA, and may have a prosurvival effect, further highlighting the fine balance between UPR^ER^ signalling in cell homeostasis and death (Hetz *et al*. 2006).

Besides Ire1 signalling, PERK and ATF6 activations increase the levels of the proapoptotic transcription factor CHOP, which enhances the Bcl-2 family protein Bim and promotes apoptosis (McCullough *et al*. 2001; Puthalakath *et al*. 2007) (Fig. 1). UPR^ER^-triggered cell death pathways were shown to be anti-viral, and limit virus dissemination, their effects were not investigated in detail.

**Figure 5.**
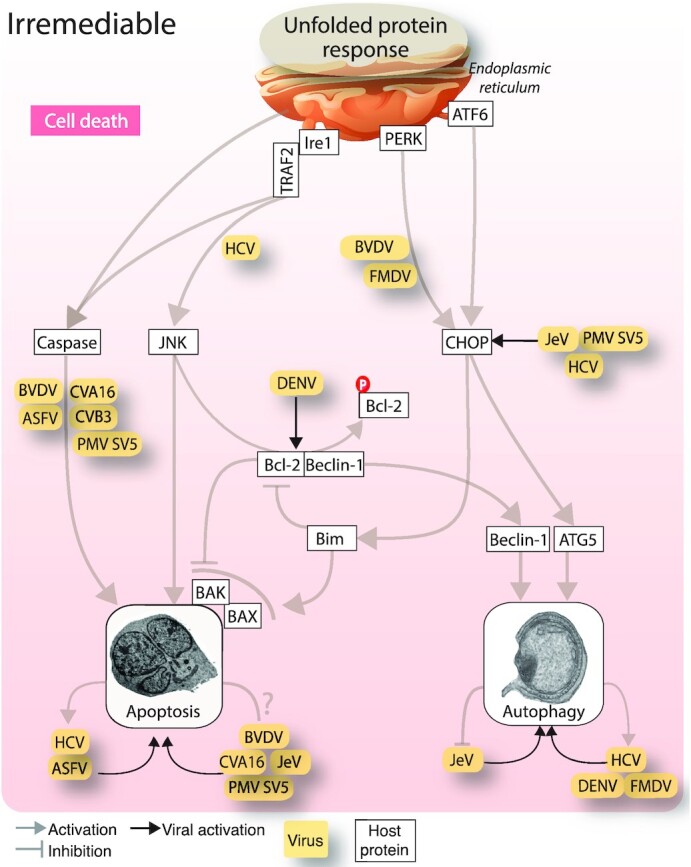
Viral modulation of UPR^ER^ controlled cell death pathways. Viruses and viral proteins use all the three UPR^ER^ sensor proteins for modulating cell death. The Ire1α-TRAF2-JNK signalling arm is used to promote apoptotic cell death. Several viruses promote ER stress dependent apoptosis via the caspase cascade. The PERK and ATF6 arms of the UPR^ER^ are targeted for the induction of autophagy via the CHOP transcription factor. Abbreviations: JNK, c-Jun N-terminal kinases; Ire1, Inositol-requiring enzyme 1; PERK, Protein kinase activated by double stranded RNA (PKR)-like ER kinase; ATF6, Activating transcription factor 6; BVDV, Bovine viral diarrhea virus; HCV, Hepatitis C virus; ASFV, African swine fever virus; CVA16, Coxsackievirus A16; CVB3, Coxsackievirus B3; JeV, Japanese encephalitis virus; PMV, Paramyxo simian virus; DENV, Dengue virus; TRAF2, TNF receptor associated factor 2; Bak, Bcl-2 homologous antagonist or killer; BAX, Bcl-2 associated X-protein; Bim, Bcl-2 like protein 11.

Below we list examples of enveloped and non-enveloped viruses that induce UPR^ER^ associated apoptosis. For details, see also Fig. 5.

***African swine fever virus (ASFV)***—Increased caspase 3, 9 and 12 levels and apoptosis induction appear to be beneficial for the release of ASFV, as suggested by a reduction in virus egress from cells treated with caspase-3 inhibitors (Galindo *et al*. 2012). The enhanced caspase-12 is an ER-stress activated apoptotic response that occurs in ASFV infection primarily via ATF6 branch of the UPR^ER^ activation (Nakagawa *et al*. 2000). See Fig. 5.

***Bovine viral diarrhea virus (BVDV)***—BVDV is a positive-strand RNA virus of the genus pestivirus, and along with other members of the *Flaviviridae* family, uses the host ER as the primary site of replication and progeny assembly. BVDV infection is associated with pathogenicity and linked to the activation of PERK, as suggested by hyper-phosphorylation of eIF2α and caspase-12 meditated apoptotic cell death (Jordan *et al*. 2002).

***Coxsackievirus (CV)***—CVA16 infection showed an increase in caspase-3, 8 and 9 dependent apoptotic death. Apoptotic death was blunted by the chemical chaperone 4-methyl butyric acid, which increases the folding capacity of ER and reduces the UPR^ER^ (Zhu *et al*. 2013). The role of UPR^ER^ sensors in triggering BVDV apoptosis is unknown. In addition to the involvement of caspase-3, 8 and 9, an increase in the levels of caspase-7 and 12 and increased apoptotic cell death was observed in CVB3 infection (Zhang *et al*. 2010).

***Hepatitis C virus (HCV)***—A replicon expressing the HCV core protein in cultured cells, and the liver of transgenic HCV infected mice showed progressive depletion of ER Ca^2+^ stores and an increase in the pro-apoptotic UPR^ER^ induced factor CHOP, leading to an induction of apoptosis, possibly contributing to HCV-induced chronic liver disease (Benali-Furet *et al*. 2005). Whether PERK and ATF6 activations contributed to increased CHOP levels was not reported. Since HCV suppresses PERK signalling (Fig. 3), increased CHOP levels could at least in part be due to ATF6. Accordingly, HCV infection enhanced the UPR^ER^ via Ire1α−JNK pathway which could contribute to triggering cell death pathways (Chusri *et al*. 2016). See Fig. 2.

***Japanese encephalitis virus (JeV)***—A study reported that the UPR^ER^ induction in JeV-infected cells increased the CHOP protein levels and enhanced apoptotic death (Su *et al*. 2002). An overall UPR^ER^ might be responsible for this effect (Fig. 3).

***Paramyxo simian virus 5 (PMV SV5)—*** Apoptotic cell death was induced by PMV SV5 involving an increase in the levels of ER stress activated host factors, including BiP, CHOP, Calnexin, suggesting a contribution by the virus-induced UPR^ER^ (Sun *et al*. 2004) (Bitko and Barik 2001). The increase in cell death during SV5 infection was linked to caspase-3 and ER-stress activated caspase-12 (Fig. 5).

## THE UPR^ER^ AND ONCOLYTIC VIRUSES

Cancer cells exhibit aggravated UPR^ER^, which is part of their program to establish homeostasis in the context of the organism (Hsu *et al*. 2019). This offers a potential angle to repurpose viruses towards oncolytic therapy, if the viruses of interest normally benefit from the UPR^ER^. Several *in vivo* and *in vitro* evaluations of different oncolytic viruses in the context of the UPR^ER^ have been reported. Oncolytic viruses are engineered such that they preferentially infect and kill cancerous cells and spare the normal cells (for reviews, see Russell, Peng and Bell 2012; Alemany 2013; Gao *et al*. 2019; Georgi and Greber 2020; Lemos de Matos, Franco and McFadden 2020). Increased virus-induced cancer cell death via UPR^ER^ induction has been demonstrated in tissue culture. For example, the enhancement of UPR^ER^ by pharmacological agents targeting the early secretory pathway induced the UPR^ER^ and increased AdV gene expression and lytic cell death (Prasad *et al*. 2014). The effect occurred through the Ire1-Xbp1 axis of the UPR^ER^. On the other hand, pharmacological inhibition of Ire1α and PERK decreased autophagy and enhanced alphavirus M1 oncolytic activity in mice (Li *et al*. 2018).

Akin to oncolytic viruses, chemotherapeutic drugs modulate the tumour microenvironment and can enhance the anti-tumour immune response (Grootjans *et al*. 2016). Combining these two anti-tumour modalities has the potential to improve anti-tumour efficacy at low toxicity *in vitro* and *in vivo* (Simpson *et al*. 2016). For example, a combination therapy of ERK1/2 inhibitors enhanced the oncolytic efficiency of reovirus via induction of UPR^ER^ in murine melanoma tumours (Roulstone *et al*. 2015). Enhancement of the UPR^ER^ by the FDA-approved proteasome inhibitor Bortezomib increased oncolytic HSV-1 replication in several cancerous cell lines and also in a murine glioma cancer model (Yoo *et al*. 2014). In a murine abdominal cancer model, inhibitors against Ire1α turned out to sensitise cancer cells towards caspase-2 dependent apoptosis after rhabdovirus infection (Mahoney *et al*. 2011). Similarly, the UPR^ER^ induction by Bortezomib showed the activation of EBV lytic switch gene ZTA in Burkitt lymphoma cells via enhanced expression of CCAAT/enhancer-binding protein (C/EBP) ß (Shirley *et al*. 2011). Similarly, the UPR^ER^ inducer thapsigargin, which inhibits the ER Ca^+2^ ATPase, appeared to trigger a lytic switch, although underlying mechanisms were not identified (Shirley *et al*. 2011). The fine balance between homeostasis and apoptotic induction by the UPR^ER^, now requires more mechanistic knowledge of virus interactions with the UPR^ER^, and drug synergy experiments, before this field is ripe for clinical applications.

## EVOLUTIONARY IMPACT OF VIRUSES ON THE UPR^ER^

### UPR^ER^ sensors in the context of cell stress

So far, we have discussed evidence for virus interactions with the UPR^ER^, and how the UPR^ER^ maintains cell homeostasis, survival and the onset of death processes in virus infections. Other interconnections of the UPR^ER^ to cell stress pathways are emerging, such as anti-viral response signalling, innate immunity through both cytokines and intrinsic factors, reactive oxygen signalling and metabolic pathways (Rouse and Sehrawat 2010; Wolfrum and Greber 2013; Thaker, Ch'ng and Christofk 2019). For example, the NLRP3-caspase-2 dependent signalling can integrate the UPR^ER^ and innate immunity and relay it to the mitochondria to promote inflammation (Bronner *et al*. 2015; Lencer *et al*. 2015). ER stress also regulates innate and adaptive immune responses, including NF-κB signalling and the type I IFN response (Liu *et al*. 2012). Oxidative stress further triggers the UPR^ER^ in vertebrates, and installs a feed-forward loop to remedy oxidative damage (Schwarz 1996). This is akin to the activation of Ire1-Hac1 in yeast, which upregulates genes protecting cells from oxidative stress, such as TSA1, a thioredoxin peroxidase catalyzing the reduction of peroxides and acting as a molecular chaperone (Kimata *et al*. 2006).

These signalling networks interlinking with the UPR^ER^ provide rich opportunities to explore evolutionary adaptation. For example, viruses toggle switch between lytic and persistent infection outcomes, as shown with herpes viruses and B-cell receptor signalling through Xbp1, which triggers the reactivation of latent KSHV into a lytic cycle (Kati *et al*. 2013; Johnston and McCormick 2019). Evolutionary impact of KSHV and other viruses, such as EBV, on the UPR^ER^ may have facilitated the development of vertebrate pathways to synthesize large loads of protein, for example immunoglobulins from B cells or collagen from notochord cells during development (Sun and Thorley-Lawson 2007; Ishikawa *et al*. 2017; Mrozek-Gorska *et al*. 2019). In addition, remodeling of ER membranes during virus infections to set up membranous webs or membrane zippering may further contribute to evolutionary adaptation of the ER during global UPR^ER^ (Sriburi *et al*. 2004; Romero-Brey and Bartenschlager 2016).

An evolutionary impact of viruses on the vertebrate UPR^ER^ may occur through sensors detecting both protein and lipid stress (Kono, Amin-Wetzel and Ron 2017; Preissler and Ron 2019). Both Ire1α and PERK are dual sensors, which may recognize specific features in pathogenic processes. For example, the AdV E3-19K glycoprotein specifically activates the Ire1α branch without inducing RIDD or cell death pathways (Prasad *et al*. 2020). Evolutionary tuning of the UPR^ER^ by viruses is further supported by the observation that viral proteins utilize both direct binding to the sensors or sequestration of BiP away from the sensors to trigger a UPR^ER^ response (Perera, Miller and Zitzmann 2017; Prasad *et al*. 2020). Such dual modality in sensor activation has recently been simulated in mathematical models, and was suggested to best account for the full activation spectrum of the UPR^ER^ (Stroberg, Eilertsen and Schnell 2019).

### Yeast Ire1-mediated mobilization of retroelements is disabled in vertebrates

In vertebrates, the UPR^ER^ is a conserved three-pronged stress response that primarily restores homeostasis, where Ire1 is its most conserved arm. Lower eukaryotic cells have less complex UPR^ER^ pathways. The simple eukaryote *Saccharomyces cerevisiae* has just one ER stress sensor, yIre1 (Cox, Shamu and Walter 1993; Mori *et al*. 1993). yIre1 is thought to directly bind to unfolded proteins in the ER lumen and activate its cytosolic RNase domain leading to splicing of the precursor HAC1u to the mature HAC1i mRNA, which is translated to the functional transcription factor Hac1p (Mori *et al*. 2000; Ruegsegger, Leber and Walter 2001). Intriguingly, functional genetics studies in *S. cerevisiae* indicated that the dormancy of transposable elements can be modulated by the UPR^ER^, and, in addition, by a variety of extrinsic and intrinsic cues, including stress signalling through mitogen-activated kinases, DNA damage, environmental signals such as temperature and nutrient availability (Carr, Bensasson and Bergman 2012).

We surmise that the large abundance of retroviruses in vertebrates has disabled a modality of the Ire1-Xbp1, such that Ire1 no longer activates genomic retrotransposons. Specifically, one of the two major retrotransposons in *S. cerevisiae*, Ty2 is transcriptionally upregulated by the yIre1-Hac1 pathway of the UPR, as shown in cDNA microarray analyses and knock-out strains (Kimata *et al*. 2006). HAC1 mutagenesis generated cells which expressed Hac1i mRNA without Ire1 activation, and these cells induced Ty2 transcription, indicating the importance of the primordial Ire1 UPR^ER^ arm in controlling retroelements (Kimata *et al*. 2006). Importantly, *S. cerevisiae* responds to the potent ER stressors dithiothreitol and tunicamycin by upregulating UPR^ER^ target genes, including those involved in ERAD, intracellular vesicle transport and lipid biosynthesis, and downregulating genes encoding proteins destined to the secretory pathway (Travers *et al*. 2000; Kimata *et al*. 2006). Although vertebrate genomes harbor large amounts of transposable elements, most of these elements are inactive and strongly suppressed, whereas yeast cells, which have only a few genome % retroelements, show high levels of retrotransposition events (Curcio, Lutz and Lesage 2015; Sotero-Caio *et al*. 2017). This argues for a very tight control of retroelements in vertebrate cells. In fact, retrotransposons dramatically enhance alterations in the genome by mediating chromosomal rearrangements, including deletions, segmental duplications, inversions and reciprocal and non-reciprocal translocations (Curcio, Lutz and Lesage 2015).

The Ty elements are the evolutionary progenitors of retroviruses in vertebrates. Transcription of these elements is initiated at the 5′ LTR and gives rise to an RNA from which the element-encoded proteins Gag and Gag-Pol are translated, comprising protease, integrase, reverse transcriptase and RNase H. Together with host proteins, Gag-Pol are assembled into virus-like particles, processed by the protease, and thereby serve as essential replication intermediates for the reverse transcription of the RNA into DNA and genomic insertion. The introduction of Ty2 retroelements into an ancestor of *S. cerevisiae* occurred rather recently as a result of horizontal transfer, whereas the Ty1 elements, which no longer respond to the UPR^ER^, are more ancient (Kimata *et al*. 2006; Carr, Bensasson and Bergman 2012; Curcio, Lutz and Lesage 2015). It thus appears that the response of retrotransposons to the Ire1-Xbp1/Hac1 activation pathway declines in the course of evolution, and the decline correlates with increased genomic load of retroelements. One could argue that the high abundance of mobile genetic elements, including endogenous and exogenous retroviruses in vertebrates, selects for cells that no longer use the UPR^ER^ for boosting genomic rearrangements by their retroelements.

The proper control of retrotransposons is crucial for cell and organismic survival. For example, the impaired silencing of retrotransposons has been shown to trigger the excessive expression of retroviral env glycoproteins and thereby activate a general UPR^ER^, causally linked to increased pro-B cell death through inactivation of the epigenetic regulator Setdb1 and an increase in histone H3-lysine 4 trimethylation (Pasquarella *et al*. 2016). This phenotype is exacerbated by the expression of enhanced levels of double-stranded RNA from endogenous retroviruses, and by triggering pattern-recognition receptors, such as RIG-I, and IFN (Roulois *et al*. 2015). We surmise that endogenous retroviruses exert evolutionary force on the cell death pathways of UPR^ER^ and synergize with interconnected innate immunity pathways to reach organismic homeostasis. This supports the possibility that the UPR^ER^ coevolved with multicellular eukaryotes, where cells of the immune system have adopted specialized functions requiring adaptations of the ER and its UPR.

## CONCLUSIONS AND OUTLOOK

Viruses have a long history of inducing stress responses in their hosts. Stress responses are multifaceted and interconnected, and are accessible to tuning by the pathogens. They are evolutionary conserved, and reach back to bacterial cells, where phages increase the levels of heat shock proteins to restore homeostasis upon stress insult (Drahos and Hendrix 1982; Young 1990). Together with the pathogen, stress responses define the outcome of the infection, cytoprotective or cytotoxic. The UPR^ER^ is a significant eukaryotic stress response, controlling cell survival or death. In communicating with other signalling pathways it modulates innate immune and metabolic responses. This review illustrated how the UPR^ER^ is triggered by viruses, and how viruses overcome the antiviral effects of the UPR^ER^. A deeper understanding of how viruses interact with the UPR^ER^ will require more mechanistic studies and also evolutionary insights. Chemical genetics, small molecule modulators of the UPR^ER^, and the power of virology will provide the field with new opportunities not only for developing anti-viral interference but also for therapies against nonviral diseases exacerbated by the UPR^ER^. For example, we envision that lipid homeostasis and the UPR^ER^ in viral infections will be important to understand how viruses switch between lytic and nonlytic propagation. Notably, key enzymes of lipid metabolism locate to the ER and make this organelle a primary hub for both structural and metabolic components in cell homeostasis.
